# Diminished cytokine-induced Jak/STAT signaling is associated with rheumatoid arthritis and disease activity

**DOI:** 10.1371/journal.pone.0244187

**Published:** 2021-01-14

**Authors:** Jason Ptacek, Rachael E. Hawtin, Dongmei Sun, Brent Louie, Erik Evensen, Barbara B. Mittleman, Alessandra Cesano, Guy Cavet, Clifton O. Bingham, Stacey S. Cofield, Jeffrey R. Curtis, Maria I. Danila, Chander Raman, Richard A. Furie, Mark C. Genovese, William H. Robinson, Marc C. Levesque, Larry W. Moreland, Peter A. Nigrovic, Nancy A. Shadick, James R. O’Dell, Geoffrey M. Thiele, E. William St Clair, Christopher C. Striebich, Matthew B. Hale, Houman Khalili, Franak Batliwalla, Cynthia Aranow, Meggan Mackay, Betty Diamond, Garry P. Nolan, Peter K. Gregersen, S. Louis Bridges

**Affiliations:** 1 Nodality, Inc., South San Francisco, California, United States of America; 2 University of Alabama at Birmingham School of Medicine, Birmingham, Alabama, United States of America; 3 Johns Hopkins University School of Medicine, Baltimore, Maryland, United States of America; 4 The Feinstein Institute for Medical Research and Northwell Health, Manhasset, New York, United States of America; 5 Stanford University School of Medicine, Stanford, California, United States of America; 6 Abbvie, Inc., North Chicago, Illinois, United States of America; 7 University of Colorado Anschutz Medical Campus, Boulder, Colorado, United States of America; 8 Brigham and Women’s Hospital, Harvard University, Boston, Massachusetts, United States of America; 9 University of Nebraska Medical Center, Lincoln, Nebraska, United States of America; 10 Duke University Medical Center, Durham, North Carolina, United States of America; 11 Hospital for Special Surgery and Weill Cornell Medical College, New York, New York, United States of America; Western Kentucky University, UNITED STATES

## Abstract

Rheumatoid arthritis (RA) is a systemic and incurable autoimmune disease characterized by chronic inflammation in synovial lining of joints. To identify the signaling pathways involved in RA, its disease activity, and treatment response, we adapted a systems immunology approach to simultaneously quantify 42 signaling nodes in 21 immune cell subsets (e.g., IFNα→p-STAT5 in B cells) in peripheral blood mononuclear cells (PBMC) from 194 patients with longstanding RA (including 98 patients before and after treatment), and 41 healthy controls (HC). We found multiple differences between patients with RA compared to HC, predominantly in cytokine-induced Jak/STAT signaling in many immune cell subsets, suggesting pathways that may be associated with susceptibility to RA. We also found that high RA disease activity, compared to low disease activity, was associated with decreased (e.g., IFNα→p-STAT5, IL-10→p-STAT1) or increased (e.g., IL-6→STAT3) response to stimuli in multiple cell subsets. Finally, we compared signaling in patients with established, refractory RA before and six months after initiation of methotrexate (MTX) or TNF inhibitors (TNFi). We noted significant changes from pre-treatment to post-treatment in IFNα→p-STAT5 signaling and IL-10→p-STAT1 signaling in multiple cell subsets; these changes brought the aberrant RA signaling profiles toward those of HC. This large, comprehensive functional signaling pathway study provides novel insights into the pathogenesis of RA and shows the potential of quantification of cytokine-induced signaling as a biomarker of disease activity or treatment response.

## Introduction

Rheumatoid arthritis (RA) is a heterogeneous, chronic systemic disease characterized by synovial inflammation, cartilage destruction, and progressive bony erosions, often leading to joint deformity and disability with significant associated organ system involvement [1]. While the cause is unknown, RA is characterized by dysregulation of immune responses through the production of autoantibodies and cytokines. Many immune cells such as monocytes, T and B cells are known to play essential roles in RA pathogenesis [2]. Dysregulation of intracellular signaling pathways involving Jak/STAT signaling in different circulating immune cell subsets is thought to mediate the chronic inflammatory response [3–6]. A recent analysis of single-cell transcriptomics and mass cytometry from RA joint synovial tissues confirmed the critically important role of activated immune cells and fibroblasts, increased cytokine expression and of provocative pathways, especially Jak/STAT signaling, in the synovitis that defines RA [7].

Conventional small molecule disease-modifying anti-rheumatic drugs (DMARDs) are first line treatments of RA, with MTX as the anchor drug and typically the first DMARD started for active RA. In the late 1990s, biologic medications such as those inhibiting TNF, IL-6, IL-1, as well as those targeting B cells and T cells became commonly used to effectively treat RA. The latest class of drugs, targeted small molecules, include three JAK/STAT inhibitors: tofacitinib, baricitinib, and upadacitinib. These drugs preferentially inhibit JAK-1 and JAK-3; JAK-1 and JAK-2; and JAK-1, respectively. Although MTX is the most effective conventional DMARD in RA, only about 30% of patients with early RA achieve low disease activity within 6 months [8]. Despite the introduction of biologic drugs and targeted small molecules, fewer than half of patients with RA reach 70% improvement in disease activity as defined by the American College of Rheumatology (ACR) response criteria (ACR70) [1].

The mechanism by which MTX acts to reduce disease activity remains under active investigation. It is a known inhibitor of dihydrofolate reductase but does not likely work in RA through that mechanism as folate supplementation during MTX therapy lessens adverse events but does not diminish its effectiveness [9]. At present, it is thought that the major mechanism of action of MTX in RA involves the adenosine pathway [10]. However, there are several lines of emerging evidence that MTX may improve disease activity in RA through effects on JAK/STAT signaling pathways. Screening of a library of 2,000 small molecules using a cell-based Drosophila JAK/STAT reporter assay identified MTX as a JAK/STAT inhibitor [11]. In addition, MTX reduces STAT1 and STAT5 phosphorylation levels in human Hodgkin’s lymphoma-derived cell lines [12].

TNF inhibitors were the first category of biologic DMARDs to be widely used in RA. TNF is produced by activated macrophages, lymphocytes and natural killer (NK) cells and is a major inflammatory cytokine found in synovium of patients with RA. Although it is known that TNF blockade prevents the binding to TNF receptors and activation of downstream signaling [13], the precise mechanisms of action underlying their efficacy remain unknown. *In vitro* studies of the TNF inhibitor adalimumab showed that it promoted IL-10 expression in CD4+ T cells and delayed T-cell activation and maturation [14]. Anti-TNF treated CD4+ T cells also displayed a reduced ability to induce IL-6 and IL-8 production by synovial fibroblasts. There are no robust, widely used biomarkers to predict clinical response to MTX or TNFi, which may reflect incomplete knowledge of the cellular and molecular pathways through which these drugs exert their therapeutic effect and represents a large knowledge gap in RA.

Investigations into the pathologic roles of peripheral blood immune cells in RA have typically focused on 1–3 signaling pathways in a limited number of cell subsets due to technical challenges of obtaining sufficient numbers of cells to analyze multiple pathways in many cell subsets simultaneously. In this study, we use single cell network profiling (SCNP), a phosphoflow cytometry method that allows simultaneous interrogation of many basal (unstimulated) and exogenously stimulated intracellular signaling pathways in multiple immune cell subsets within a single aliquot of PBMC [15, 16]. This technique makes it possible to simultaneously analyze multiple signaling pathways in numerous cell subsets and has been applied to characterize immune signaling profiles in order to identify prognostic or predictive biomarkers in several diseases including acute myeloid leukemia [17, 18].

In this hypothesis-generating analysis we used SCNP to study 42 signaling nodes, chosen for their reported central roles in RA pathobiology, in 21 immune cell subsets in patients with RA compared to HC. Here we report our most important findings, largely focused on four cytokine-induced Jak/STAT signaling pathways in multiple immune cell subsets (subsets of T cells, B cells and monocytes). We assess the roles of these pathways in RA susceptibility (comparing RA to HC), disease activity (stratified by DAS28), and response to treatment with MTX or TNFi. Our study provides novel insights into which cytokine-induced Jak/STAT signaling pathways and cell subsets are important in the pathogenesis of RA. Furthermore, these findings may lead to the development of cellular biomarkers to assess the underpinnings of successful treatment response, thus improving the quality of life and cost-effectiveness of RA patient management.

## Materials and methods

### Human subjects

PBMC from a total of 48 patients with RA were collected and cryopreserved at North Shore Long Island Jewish Health System (Cohort1). These subjects all had longstanding RA and were on active treatment with various combinations of drugs such as MTX and biologic agents. PBMC from 20 age and sex-matched HC were collected based on an IRB-approved protocol at School of Medicine of Stanford University.

A second group of RA patients was from the NIH-funded TETRAD Study (see ClinicalTrials.gov link: https://clinicaltrials.gov/ct2/show/NCT01070121?term=University+of+alabama+at+Birmingham&cond=Rheumatoid+Arthritis&cntry=US&state=US%3AAL&city=Birmingham). TETRAD was an NIH-funded observational study which enrolled 200 RA patients from nine academic medical centers in the US. Inclusion criteria were: age > 19 years; fulfillment of the 1987 ACR classification criteria for RA; and initiating one of the following DMARD or biologic therapies for clinically indicated reasons: MTX, TNFi (adalimumab, etanercept, golimumab, or infliximab), abatacept, rituximab, or tocilizumab. A total 146 patients’ samples were included in the baseline pre-treatment analyses (TT0) and 98 patients’ samples were included in the follow up analyses 6 months after initiating index treatment (T6M). HC (n = 10 for TT0, n = 11 for T6M) were obtained from demographically matched donors from the Stanford Blood Bank. High quality prospective clinical data on disease activity (DAS28) and treatment response (change in DAS28) were collected for each patient. PMBC were isolated and cryopreserved as described previously [19]. All patients’ samples were collected under study protocols approved by the local IRBs at each institution. The institutions include University of Alabama at Birmingham (IRB00000196, FWA00005960), North Shore Long Island Jewish Health System (09–303), Johns Hopkins University (NA_00034921), University of Colorado (10–0270), University of Pittsburgh (PRO09110503), University of Nebraska (022-10-FB), Stanford University (6208), Duke University (Pro00022649), Brigham and Women’s Hospital/Harvard University (FWA00003136). In accordance with the Declaration of Helsinki, all patients provided written informed consent for the collection and use of their blood samples for research purposes.

### Single cell network profiling (SCNP) assay

The SCNP assay was performed for all studies following the general experimental methods described previously [1, 17, 19, 20]. We designated a stimulus and readout as a node. For example, IFNα stimulated phosphorylation of STAT1 is designated as “IFNα→p-STAT1”. The “metric” is the quantitative evaluation of signaling protein response, as described previously [21, 22], where a zero indicates no induced signaling and positive values indicate increases in signaling. Median fluorescence intensities (MFIs) were obtained from cell fluorescence intensity levels for all samples; the raw data were converted to plate-calibrated Equivalent Number of Reference Fluorophores (ERFs) using rainbow calibration particles (Spherotech). In this study, we used the log2Fold metric to measure the magnitude of the responsiveness compared to unstimulated cells using the formula:

$${\text{log}}_{2}({\text{ERF}}_{\text{stimulated}}/{\text{ERF}}_{\text{unstimulated}}).$$

Cryopreserved PBMC samples were thawed, washed, resuspended in RPMI 1640 (10% FBS), aliquoted to 96-well plates at 1 million cells (Cohort 1) or 100,000 cells (TT0, T6M) per well and rested for 2 hours at 37°C prior to stimulation. Propidium iodide (PI) staining (Cohort 1) or Amine Aqua viability dye (TT0, T6M) were used to distinguished non-viable cells. Following stimulation (S1 Table) for 2 to 30 minutes (depending on the biologic pathway being assayed), the cells were fixed with PFA, permeabilized with ice-cold methanol, and stored at -80°C as previously reported [23]. To assess more nodes with limited patient material, the cytokines IL-2 and GM-CSF were used in combination to interrogate STAT5 signaling: the non-overlapping cell specificity of these cytokines enabled interrogation of IL-2 induction of STAT5 signaling in T cells, and GM-CSF induction of STAT5 signaling in monocytes. Cells from all cohorts were washed in FACS Buffer (PBS, 0.5% BSA, 0.05% NaN_3_), stained with cocktails of fluorochrome-conjugated antibodies (S2 Table), and analyzed on LSR II (Cohort 1) or CANTO II (TT0, T6M) flow cytometers using FACS DIVA software (BD Biosciences). All flow cytometry data were analyzed with FlowJo (TreeStar Software, Ashland, OR) or WinList (Verity House Software, Topsham, ME).

The SCNP assay incorporates a number of standardized procedures and process controls that include instrument standardization and calibration, reagent qualification and quality control testing, consistent sample processing, and assay performance monitoring [21]. For TT0 and T6M sample processing, a cell line control row (consisting of the GDM-1, Ramos, or Jurkat cell lines arrayed according to the biology tested, e.g. Jurkat cells as a control for TCR stimulation) were included in each of the 96-well plates that were processed. In addition, a “bridging control” donor PBMC sample was included with each batch of samples. The controls were used to monitor the reproducibility of the assay performance. Dead cells and debris were excluded by forward scatter (FSC) and side scatter (SSC) for all samples; for TT0 and T6M, cPARP staining was also included to remove dying cells from the analyses. Gating strategies to delineate cell populations are shown in S1 Fig and S3 Table.

### Outcome measurement

Our outcomes of interest are: 1) the presence versus absence of RA (we compare RA to HC to gain insight into factors associated with susceptibility to RA); 2) disease activity at a single point in time (we compare patients with various levels of active synovitis and inflammation); and 3) treatment response (we compare a group of patients before and after treatment with MTX or TNFi).

To determine which signaling pathways are associated with RA, we applied SCNP to RA and HC samples from Cohort 1, TT0 and T6M. To study signaling pathways associated with RA disease activity, we compared SCNP profiles from RA patients (TT0) who were starting MTX or TNFi for clinically indicated reasons. DAS28 was used to determine disease activity of Cohort 1, baseline TT0 and follow up T6M (6 months after initiation of treatment;). The DAS28 score is a continuous score that describes the degree of activity of RA. The number of swollen joints and tender joints were counted using the 28 version of simplification of original 44 joints score and the ESR, a measure of inflammation, was assayed in the local lab of each patient. We used the DAS28 with 3 variables to calculate the Modified Disease Activity Score (DAS) as follows: (0.56*sqrt (TENDER) + 0.28*sqrt (SWELL) + 0.70*ln (ESR))*1.08 + 0.16. The DAS28 score can be used to categorize patients into those with high (> 5.1), moderate (3.2 < DAS28 ≤ 5.1), or low disease activity (2.6 < DAS28 ≤ 3.2) or remission (< 2.6) [24].

### Data analysis

Specific statistical tests used for each analysis are specified in the figure legends. Spearman’s correlation analysis was used to test the association of signaling protein phosphorylation with disease activity. The two-sample Wilcoxon’s rank sum test and non-parametric Kruskal-Wallis test were adapted to test the difference in signaling responses between HC *vs* RA patients or multiple groups with GraphPad Prism 7, if applicable. P-values <0.05 were considered statistically significant.

### Study approval

Patient samples were collected under study protocols approved by the local IRBs at each institution. In accordance with the Declaration of Helsinki, all patients provided written informed consent for the collection and use of their blood samples for research purposes.

## Results

### Consistent attenuation of cytokine induced Jak/STAT signaling in patients with RA

We studied a total of 194 RA patients: 48 from the North Shore Long Island Jewish Health System (Cohort 1) and 146 from the Treatment Efficacy and Toxicity in RA Database and Repository (TETRAD) study. TETRAD enrolled patients with longstanding RA who were about to initiate treatment with either MTX or a biologic drug (index drugs for the study) for clinically indicated reasons. Data and samples from the TETRAD study have been used to analyze patients before (TT0) and after (T6M) treatments [16, 25–27]. The majority of the drug started in TETRAD were either MTX or a TNFi, with small numbers of patients starting other drugs such as abatacept, rituximab, or tocilizumab. We focused our analysis on RA patients starting MTX or TNFi, due to insufficient statistical power to analyze the effect of other individual drugs on signaling pathways. A group of HC with similar demographic characteristics to each of the three RA groups (Cohort 1, TT0, T6M) (Table 1) were also studied.

**Table 1:** Demographic and clinical characteristics of patients of RA and HC.

	Cohort 1		TT0		T6M	
	HC (n = 20)	RA (n = 48)	HC (n = 10)	RA (n = 146)	HC (n = 11)	RA (n = 98)
**Age at Baseline (median years, range)**	55.0 (42–80)	61.5 (37–87)	49.0 (40–60)	56.0 (24–82)	45 (29–60)	56.0 (25–80
**Sex (n, % female)**	14 (70%)	40 (83.3%)	6 (60%)	126 (86.3%)	7 (63.3%)	83 (84.7%)
**Race/Ethnicity (n, %)**						
Caucasian	10 (50%)	N.A.	5 (50%)	113 (77.4%)	5 (50%)	79 (80.6%)
Asian American	3 (15%)	N.A.	2 (20%)	8 (5.5%)	2 (20%)	6 (6.1%)
African American	0 (0%)	N.A.	0 (0%)	16 (10.9%)	0 (0%)	8 (8.2%)
Other/Unknown	7 (35%)	48 (100%)	3 (30%)	9 (6.2%)	3 (30%)	5 (5.1%)
**Anti-CCP status (n, %)**						
Positive	N.A.	31 (64.6)	N.A.	91 (62.4%)	N.A.	62 (63.3%)
Negative	N.A.	17 (35.4%)	N.A.	34 (23.2%)	N.A.	22 (22.4%)
Unknown	N.A.	0	N.A.	21 (14.4%)	N.A.	14 (14.3%)
**RF status (n, %)**						
Positive	N.A.	41 (85.4%)	N.D.	103 (70.5%)	N.A	66 (67.4%)
Negative	N.A.	7 (14.6%)	N.D.	42 (28.8%)	N.A.	31 (31.6%)
Unknown	N.A.	0	N.D.	1 (0.7%)	N.A.	1 (1.0%)
**DAS28 (Median, range)**	N.A.	3.36 (1.23–6.93)	N.A.	4.96 (1.25–8.22)		3.50 (0.16–7.52)
**Concomitant meds**						
GC	N.A.	7 (14.6%)	N.A.	29 (19.8%)	N.A.	21 (21.4%)
MTX	N.A.	21 (43.8%)	N.A.	32 (22.0%)	N.A.	35 (35.7%)
GC + MTX	N.A.	12 (25.0%)	N.A.	59 (40.4%)	N.A.	18 (18.4%)
None/Unknown	N.A.	8 (16.7%)	N.A.	26 (17.8%)	N.A.	24 (24.5%)
**Recent Previous Biologic at Baseline (n, % No)**	N.A.	N.A.	N.A.	108 (74.0%)	N.A	N.A.
**Current Biologic (n, %)**						
Abatacept	N.A.	23 (47.9%)	N.A.	N.A.	N.A.	13 (13.3%)
TNF inhibitor	N.A.	2 (4.2%)	N.A.	N.A.	N.A.	40 (40.8%)
Rituximab	N.A.	2 (4.2%)	N.A.	N.A.	N.A.	1 (1.0%)
Tolicizumab	N.A.	0	N.A.	N.A.	N.A.	19 (19.4%)
None	N.A.	21 (43.7%)	N.A.	N.A.	N.A.	25 (25.5%)

HC–healthy controls. N.A.–not applicable or not available. DAS28 –Disease Activity Score on 28 joints. GC–glucocorticoids. Anti-CCP–anti citrullinated peptide antibody. RF–rheumatoid factor. MTX–methotrexate.

**Fig 1:**
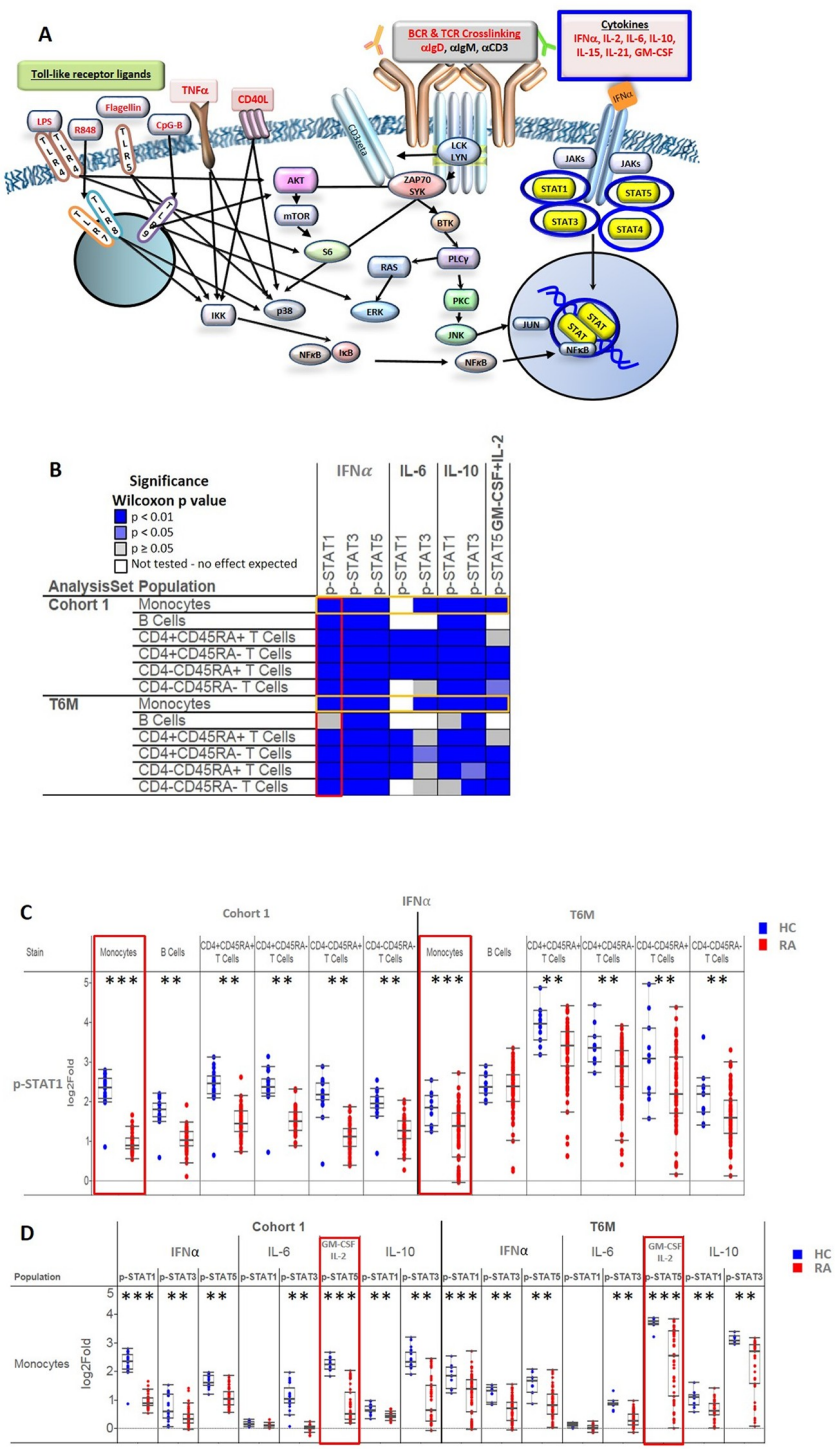
Signaling pathways relevant to the RA biology and cytokine-induced signaling analyzed in the study. A. Signaling pathways relevant to RA pathogenesis that were evaluated by SCNP. A total of 16 modulators (stimuli) were studied including cytokines that induce Jak/STAT signaling, B cell and T cell receptor crosslinking, and Toll-like receptor signaling. The cytokine-induced Jak/STAT signaling pathways examined across three cohorts and the primary focus of the report are highlighted in blue circles or boxes. Sixteen stimuli are highlighted in red text, and phosphorylated STATs examined are shown in yellow. B. Widespread reductions in *ex vivo* cytokine responses in 6 immune cell subsets are a signature of RA in Cohort 1 (n = 48 RA and n = 20 HC) and T6M (n = 98 RA and n = 11 HC). The heatmap shows significant differences in signaling between RA patients and HC. Detailed results of the experiments in nodes outlined in yellow are shown in Fig 1C. Detailed results of the experiments in nodes outlined in red are shown in Fig 1D. The pair-wise Wilcoxon signed-rank test was used, and levels of statistical significance compared to HC are shown as dark blue (p < 0.01), light blue (p < 0.05) or gray (p≥ 0.05, not significant). White boxes show cell populations that do not respond to the stimulus tested and thus were not part of this analysis. C. Representative results of analyses of the IFNα→p-STAT1 node in 6 immune cell subsets. The most significant differences between RA and HC were in IFNα→p-STAT1 in monocytes (red boxes). D. Representative results of analyses of cytokine-induced signaling in monocytes. The most significant differences between RA and HC were in GM-CSF + IL-2→p-STAT5 (red boxes). For C and D, each dot represents one subject, with RA patients shown as red dots and HC shown as blue dots. Boxes indicate the first, second (median), and third quartiles with the whiskers extending to 1.5 interquartile range (IQR). ** Differences between RA and HC were statistically significant (Wilcoxon signed-rank test) at p<0.01. *** Differences between RA and HC were statistically significant at p<0.001.

Fig 1A represents signaling nodes relevant to the immunobiology of RA gleaned from the literature. Overall, we studied 42 signaling nodes, including 16 stimuli (S1 Table) with associated intracellular phosphorylated readouts (see antibodies used in S2 Table) including STATs, AKT, ERK, etc.) in 21 immune cell subsets (see S1 and S2 Figs, and S3 Table). Due to limited availability of effector CD4+ T cells, only 20 of 21 nodes were included in the analysis. Of the many pathways analyzed, we found that IFNα, IL-6, IL-10 and GM-CSF-induced Jak/STAT signaling pathways were significantly associated with RA, in multiple cell subsets as shown graphically in Fig 1A (highlighted in blue) [1, 3]. Thus, our paper focuses on these findings.

We initially compared stimulated (IFNα, IL-6, IL-10, GM-CSF + IL-2) *ex vivo* signaling between RA patients (Cohort 1 and T6M) and their respective HC. The patients with RA in these two groups had similar disease duration and demographic characteristics. Importantly, Cohort 1 and T6M were also very similar with regard to RA disease activity [median DAS28 3.36 (range 1.23–6.93) for Cohort 1 and 3.50 (range 0.16–7.52) for T6M] and treatment status, with the majority taking biologic drugs (Table 1).

We found significantly reduced response to cytokines in immune cell subsets from patients with RA compared to HC. Fig 1B summarizes the significant differences between RA and HC across immune cell subsets and signaling nodes; a more comprehensive, detailed analysis of the cell signaling data is shown in S3 Fig. There were consistent differences in IFNα-induced STAT1, STAT3, and STAT5 signaling in monocytes, B cells, and T cell subsets, and many differences in response to IL-6, IL-10, and GM-CSF + IL-2. Interestingly, we found consistently *reduced* signaling capacity in RA in response to these stimuli, with the majority of these differences meeting a stringent threshold for statistical significance (p value < 0.01).

IFNα activation of STAT1 was significantly lower (p < 0.01) in RA than HC (Cohort 1) in the following six subsets: total monocytes, total B cells, naïve helper Th cells (CD4+CD45RA+), memory/effector Th cells (CD4+CD45RA-), naïve Tc cells (CD4-CD45RA+), and memory/effector cytotoxic Tc cells (CD4-CD45RA-) (Fig 1B and 1C). These cell subsets are important barriers of human immune system and clustered in the synovial tissue [7] of RA patients. In particular, naïve Th cells help to generate immune responses through producing cytokines in response to antigens presented by antigen-presenting cells. Memory/effector Th cells provide rapid recall (memory) repeat exposures to antigen. Cytotoxic T cell subsets (naïve Tc cells) can kill virus-infected cells while memory/effector Tc cells recognize antigens for which there was previous exposure and help to eliminate the relevant cells [28].

Importantly, our analyses of T6M validated our findings in Cohort 1: IFNα activation of STAT1 was significantly lower in RA than HC in 5 of the 6 cell subsets listed above (the exception being total B cells). The TT0 population, in which patients were about to start MTX or a biologic drug for clinically indicated reasons (typically active disease) showed a similar pattern of reduced signaling (S4 Fig). Thus, these reductions in signaling were consistent in patients with stable treatment (Cohort 1 and T6M) as well as those with active disease about to start MTX or a biologic agent.

Of the six cell subsets in which there was reduced IFNα activation of STAT1, monocytes showed the most consistent and marked decreases across the three groups examined (red boxes in Fig 1C, p < 0.001 and S4A Fig, p < 0.0001). Additional analyses of RA monocytes demonstrated significantly attenuated signaling in 7 of 8 cytokine-STAT signaling nodes in Cohort 1, T6M (Fig 1D) and TT0 (S4B Fig). Of these 7 nodes, the most significant differences between RA and HC were in co-stimulation with GM-CSF and IL-2 using p-STAT5 for readout (p = 2.27 x 10^−21^, p = 2.14 x10^-7^ and p < 0.0001in Cohort 1, T6M and TT0, respectively) (Fig 1D, and S4B Fig). Interestingly, in RA patients but not in HC, there was clear delineation between monocytes that responded to GM-CSF+ IL-2 stimulation and those that did not (S5 Fig), suggesting the presence of distinct monocyte subsets.

The results from the three RA populations were highly similar, demonstrating the reproducibility of these findings despite differences in the patient location, technical aspects of the assay (timing, location, operator, reagents), and background treatment.

### Cytokine induced Jak/STAT signaling is associated with RA disease activity

**Fig 2:**
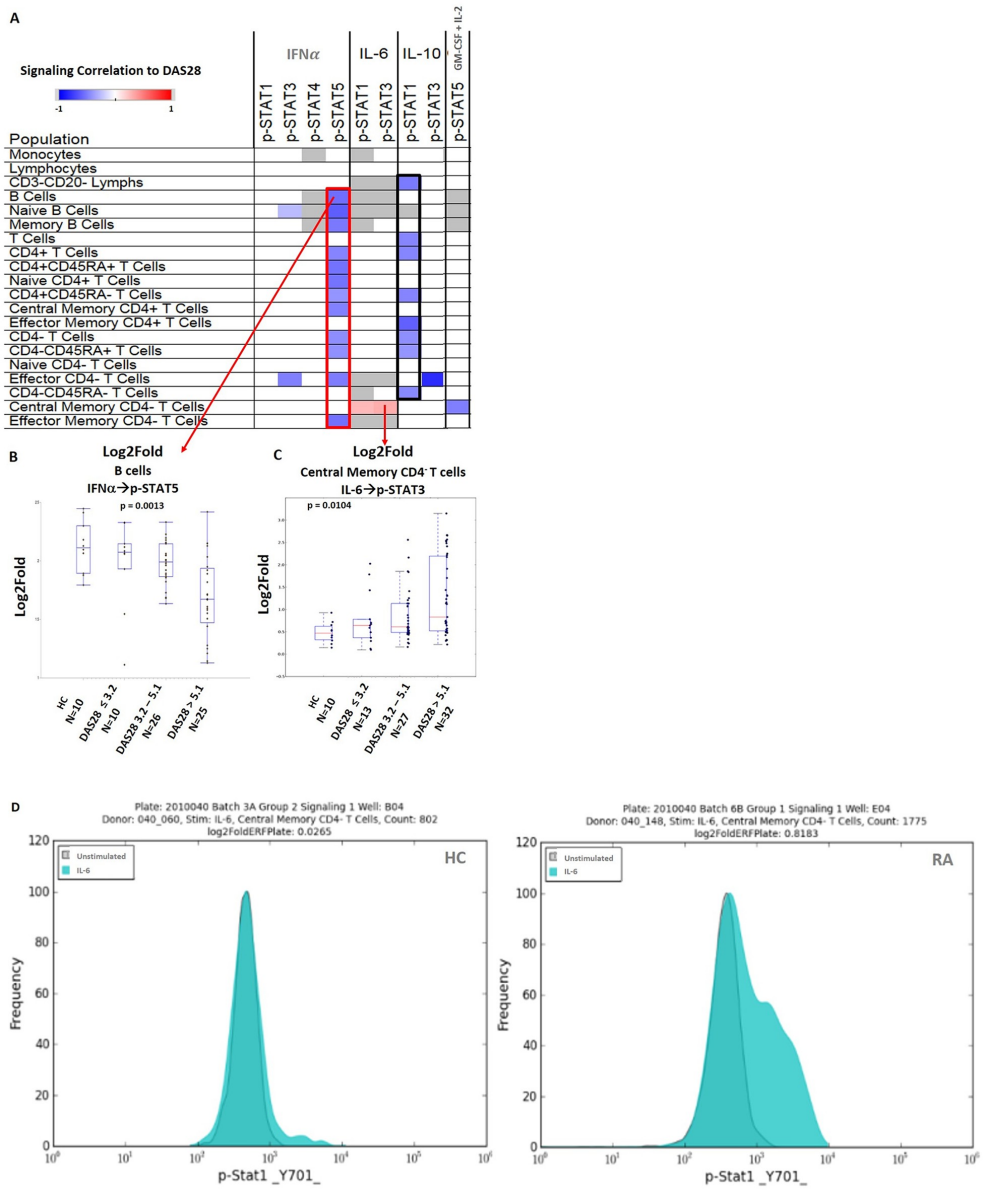
Association of signaling responsiveness with RA disease activity (DAS28). A. This heatmap shows the Spearman’s correlation (rho) between signaling response and DAS28 among TT0 patients with RA (n = 108). The correlations and scale are shown in the left upper corner. Most of the correlations were found in IFNα→p-STAT5 (outlined in red) and IL-10→p-STAT1 (outlined in black). Nodes which were not significantly associated with disease activity are shown in white. Nodes that were not expected to signal in a particular cell population (e.g. IFNα is not known to have a biologic effect on STAT4 phosphorylation) are shown in gray. Representative signaling nodes are negatively (B) or positively (C) associated with disease activity (categorized as DAS28 ≤ 3.2 (remission/low), DAS28 3.2–5.1 (moderate), or DAS ˃ 5.1(high)) in TT0, with findings from HC shown for reference. Non-parametric Kruskal-Wallis tests were used to compare groups in B and C. Boxes in B and C indicate the first, second (median), and third quantiles with the whiskers extending to 1.5 interquartile range (IQR). (D) Representative flow cytometry histograms show IL-6 stimulated (teal) compared to unstimulated (gray) p-STAT1 signaling in central memory CD4- T cells from one HC (left) and one patient with RA (right). The log2Fold metric was used to measure the magnitude of the response compared to unstimulated cells using the formula: log_2_ (ERF_stimulated_/ERF_unstimulated_).

We analyzed the TT0 dataset to find possible associations between signaling responses and the degree of RA disease activity (as assessed by DAS28 scores). TT0 was chosen for this analysis because these 108 RA patients had relatively high disease activity with a broad distribution (median DAS28 4.92, range 1.25–8.22). These patients were about to start treatment with MTX or a biologic agent, and the majority had no recent exposure to a biologic agent (Table 1). In this analysis, multiple nodes were associated (mostly inversely) with disease activity (Fig 2A). Compared to those with less active disease, RA patients with higher disease activity showed significantly less STAT5 activation in response to IFNα treatment in 12 different cell subsets (outlined in red in Fig 2A), including B cells (Fig 2B). Similarly, higher RA disease activity was associated with lower IL-10 induced STAT1 activation in 8 cell subsets (outlined in black in Fig 2A). We found only two nodes (Fig 2A) in TT0 in which higher signaling was associated with higher disease activity. These were IL-6→p-STAT1 and IL-6→p-STAT3 (Fig 2C) in central memory CD4- T cells. These findings in these two nodes in central member CD4- T cells were very similar in T6M (data not shown). Fig 2D shows representative IL-6→p-STAT1 flow cytometry plots in central memory CD4- T cells from one HC and one patient with RA. The DAS28 of the patient is 6.715 and the shift to higher levels of phosphorylated STAT1 is evident.

### Treatment with methotrexate or biologic drugs can alter cytokine induced signaling response in RA

**Fig 3:**
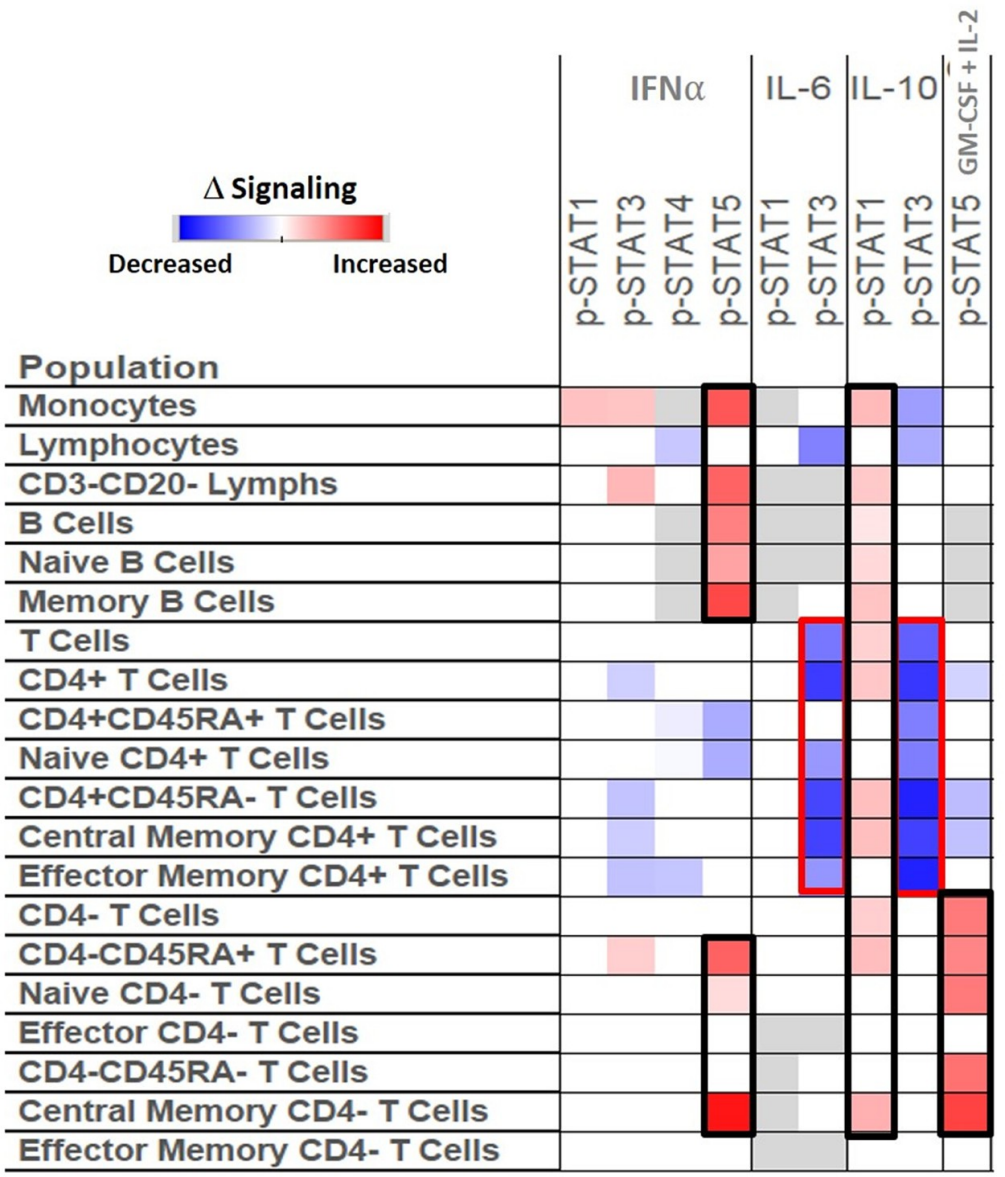
Cytokine induced signaling response is associated with disease activity changes after treatment. We compared the phosphorylation of signaling proteins in patients before (TT0) and after treatment (T6M) (median DAS28 4.83 vs 3.63, respectively) in 33 RA patients in TETRAD. The heatmap shows the median differences (log2Fold) in the signaling nodes (shown in the columns) among 20 immune cell subsets (shown in rows). Nodes that showed similar levels of signaling between the time points are shown in white. Cells that are not expected to respond to particular stimuli in specific cell populations, and were thus not included in the analysis, are shown in gray. Some of the nodes that demonstrated increased or decreased response to stimuli after treatment are highlighted in black or red, respectively.

TT0 and T6M from the TETRAD study provided a unique opportunity to explore whether the attenuated cytokine-induced Jak/STAT signaling seen in RA can be altered in immune cells after initiation of a DMARD or biologic drug. The median differences of signaling (log2Fold) in 9 nodes in 20 immune cell populations were compared between 33 RA patients before (TT0) initiation of treatment and after six months after treatment (T6M) with MTX or a TNFi. Fig 3 shows that after six months treatment, several signaling nodes in specific cell populations changed significantly, while others remained stable. Nodes that demonstrated increased response to stimuli include IFNα→p-STAT5 in monocytes, B cells, and cytotoxic T cells; and GM-CSF + IL-2→p-STAT5 in cytotoxic T cells (Fig 3). After treatment, patients’ lymphocytes (T, B, and NK cell subsets) showed less response to IL-6 stimulation, as assessed by STAT3 phosphorylation. In addition, IL-6→p-STAT3 and IL-10→p-STAT3 responses were diminished in helper T cell subsets (Fig 3, outlined in red). IL-10 stimulation induced statistically significantly greater p-STAT1 signaling in monocytes, B and T cell subsets (outlined in black) in T6M compared to TT0.

## Discussion

We performed a comprehensive analysis of basal and stimulated signaling in PBMC samples from RA patients and HC from two independent cohorts (Cohort 1 and TETRAD). We showed consistent differences between RA patients and HC in IFNα, IL-6, IL-10 and GM-CSF + IL-2 induced Jak/STAT signaling in multiple immune cell sets. We also analyzed signaling differences in RA patients with different levels of disease activity, and in paired TETRAD samples before and after initiation of treatment with MTX or TNFi.

It is unclear whether the observed diminished responsiveness in several nodes that are associated with RA is a cause, or a result of, chronic inflammation. The attenuation of induced signaling is likely not due to the effect of immunosuppressive agents, because results in TT0 (patients who were largely not recently exposed to biologic agents) were similar to those in Cohort 1 and T6M in which patients were on stable treatment with biologic agents.

We speculate that the attenuated induced signaling response in active RA is due to chronic inflammation. In support of this argument, Stone et al. recently showed that B cells “primed” in the presence of IFNγ lose the capacity, at least temporarily, to productively respond to further IFNγ triggering [29]. Whether this same mechanism is also applicable to IFNα, or more broadly applicable across other cytokines, could be the subject of further investigation. Furthermore, chronic cytokine activation in systemic lupus erythematosus (SLE, another chronic autoantibody driven autoimmune disease) is thought to render immune cells less capable of responding to stimuli [29, 30].

The importance of dysregulated B cell responses in the pathogenesis of autoimmune diseases such as RA and SLE has long been appreciated. Recent studies in SLE [31, 32] have shown that expansion of an unusual subset of B cells with a unique gene expression profile correlates strongly with disease activity. Our current findings, in the context of those from SLE, suggest that analyses of B cell signaling in more defined subsets such as DN2 (CD20^++^CD27^neg^IgD^neg^CD11c^+^) or activated naïve B cells (CD20^++^CD27^neg^IgD^+^CD11c^+^) will provide greater insight into the immunopathogenesis of RA.

In addition to associations between RA and signaling in specific pathways in specific immune cell subsets, we found important associations with RA disease activity. Furthermore, differences in signaling between T6M and TT0 changed after treatment with DMARDs such as TNFi. Interestingly, diminished signaling after *ex vivo* stimulation was altered after six months of treatment, with a trend towards the levels observed in HC. Specifically, responses of total, naïve, and memory B cells to IFNα and IL-10 stimulation (IFNα→p-STAT5, IL-10→p-STAT1) were significantly increased following 6 months of treatment with MTX or TNFi (Fig 3). This suggests a “re-setting” of the responsiveness after abrogation of the chronic inflammation *in vivo* seen in RA.

In this hypothesis-generating analysis, several nodes show promise as potential biomarkers of disease activity and treatment response. For example, lower levels of IFNα and IL-10 induced STAT5 and STAT1 activation are seen in naïve Tc cells (CD4-CD45RA+) among patients with highly active RA (Fig 2A). After six months of treatment, the levels of activation in these nodes are increased (Fig 3). Zhang et al. found that four populations of monocytes (such as interferon activated monocytes), three CD4+ clusters, three CD8+ clusters (characterized by a GZMK+, GZMB+ and GNLY+ phenotype) and four populations of B cells are expanded in RA synovial tissue and drive joint inflammation [7]. This detailed analysis of synovium, the target tissue in RA, is consonant with our observation that IFNα and IL-10 signaling differs between RA patients and HC and recovered after treatment particularly in Naïve Tc cells.

Although this study was not powered to develop and validate robust classifiers for predicting treatment response among each category of drug, the dataset provided a unique opportunity to perform exploratory analyses. We performed an exploratory analysis to look for association between baseline (pre-treatment) signaling (all signaling nodes in all cell subsets) and clinical responses at 3 months after initiation of TNFi therapy in autoantibody positive RA. Treatment responses were categorized as good, moderate, or no response (none) using European League Against Rheumatism (EULAR) criteria [33]. We initially performed a univariate analysis to identify associations of signaling with TNFi response, controlling for age and baseline DAS28 scores. Signaling nodes that were associated with TNFi response in this univariate analysis were further analyzed using an unsupervised clustering analysis. As shown in S6 Fig, a group of non-responders had elevated unmodulated signaling in seven unmodulated (unstimulated) nodes and two TNF-stimulated nodes, but low signaling in several IFNα and IL-6 stimulated nodes. In contrast, a group of good responders had opposite findings: low baseline signaling in these seven unmodulated nodes and two TNF-related nodes, with higher signaling in IFNα and IL-6 stimulated nodes. This exploratory analysis shows the potential of cell signaling as biomarkers of future treatment response to TNFi in RA.

We have previously reported on the TETRAD cohort which is analyzed extensively in this paper. We showed that in TETRAD and another RA cohort, increased circulating IFN-β/α ratio before treatment was associated with non-response to TNFi treatment [27, 34]. It is not yet clear whether TNFi treatment alters the levels of secretion of IFNα or if it affects the ability of some immune cell subsets to respond to this cytokine through JAK/STAT pathways [31, 35–37].

Our hypothesis generating observations provide additional evidence that immune cell signaling pathways are important in RA [3, 38–41], that functional immune signaling differences between RA and HC are detectable in the PBMC, and that these cell subset specific signaling pathways are possibly important in mediating clinical manifestations of RA or in the response to treatment with DMARDs or biologic agents [42–46].

Future studies could take many different directions. While the majority of our findings center on T cell subsets, there are many interesting findings among B cell subsets. We could refine our observation that responses of memory B cells to IFNα and IL-10 stimulation (IFNα→p-STAT5, IL-10→p-STAT1) were significantly increased following treatment with MTX or TNFi. This could be done by sorting and analyzing by phosphoflow cytometry CD20+CD27+IgD^neg^CD11c^neg^ cells to determine if this or other subsets within the memory B cell population that underlies the observation in this paper. Another future direction could be to identify the IFNα non-responsive B cell subsets in active RA and determine whether TNFi treatment normalizes IFNα these non-responsive B cell subsets. A final example is to determine whether IFNα-experienced B cell subsets are associated with autoantibodies such as rheumatoid factor, anti-citrullinated protein antibodies (ACPA), or anti-PAD4 antibodies, which are known to influence disease outcomes in RA [47].

## Supporting information

S1 Fig Flow cytometry gating strategy to profile immune cell subsets of the study. The gating strategy showed how to identify 21 cell subsets from PBMC with SCNP. Lymphocytes and monocytes were identified by forward scatter (FSC), side scatter (SSC). Non-viable cells were excluded based on the staining of propidium iodide (PI) staining (Cohort 1) or Amine Aqua viability dye (TT0, T6M). Lymphocytes were gated based on the expression of CD3, CD4, and CD20 to identify CD20+ B cells, CD3+CD4+ T helper cells, CD3+CD4- cytotoxic T cells, and CD3-CD20- lymphocytes that are predominantly NK cells. B cells were subdivided into naive (CD27-) and memory (CD27+) cell subsets based on CD27 expression. T helper and cytotoxic T cell populations were further subdivided into effector T cells (CD45RA+CD27-), naive T cells (CD45RA+CD27+), effector memory T cells (CD45RA-CD27-), and central memory T cells (CD45RA-CD27+).
(TIF)

S2 Fig Summary of signaling nodes examined among Cohort 1, TT0, and T6M in the comprehensive study using SCNP. A total of 42 signaling nodes (modulator → readout) in 21 immune cell subsets were evaluated with the advantage of SCNP. A core set of nodes (15 in total) and cell populations (6 in total) were analyzed in all 3 sets of samples (dark blue). In addition, due to cells availability, analyses performed in TT0 only are highlighted in yellow, analyses performed in Cohort 1 and TT0 are labeled in purple, and analyses performed in TT0 and T6M are labeled in gray. The signaling pathways of peripheral blood cells from RA patients and HC were modulated using cytokines (IFNα, IL-2, IL-6, IL-10, IL-15, IL-21, GM-CSF), crosslinking antibodies to B and T cell receptors (BCR, TCR, IgD), and TLR agonists (CD40L, TNFα, Resiquimod R848), pathogen-associated molecules (CpG-B, Flagellin and LPS) as shown on the top row. The resulting readouts measured are shown on the second row, and cell subsets analyzed are shown in the left column.
(TIF)

S3 Fig Box and whisker plots of stimulated signaling (log2Fold) in 6 immune subsets from HC and RA patients from Cohort 1 and T6M. Analyses shaded in yellow are shown in detail in Fig 1C and 1D. * Differences between RA and HC were statistically significant (Wilcoxon signed-rank test) at p<0.05. ** Differences between RA and HC were statistically significant at p<0.01. *** Differences between RA and HC were statistically significant at p<0.001.
(TIF)

S4 Fig Reduced ex vivo cytokine response in TT0 RA patients compared to HC. A. Significantly reduced IFNα→p-STAT1 signaling in 5 of 6 immune cell subsets of TT0 RA patients (n = 146) compared to HC (n = 10). * Differences between RA and HC were statistically significant (Wilcoxon signed-rank test) at p<0.05. *** Differences between RA and HC were statistically significant at p<0.001. B. Significantly reduced cytokine-induced signaling were found in monocytes of TT0 RA patients compared to HC, except IL6→p-STAT1. *** Differences between RA and HC were statistically significant at p<0.001.
(TIF)

S5 Fig Jak/STAT signaling in monocytes showed bimodal response to GM-CSF in RA compared to HC. A. Representative contour plots show p-STAT5 in monocytes from one HC and from one RA patient under three different conditions: basal (unmodulated); IFNα stimulation, and GM-CSF + IL-2 stimulation. Monocytes from the RA patients showed a bimodal GM-CSF→p-STAT5 response whereas IFNα→p-STAT5 was unimodal. B. Histograms show percentages of monocytes that respond to GM-CSF from RA patients and HC.
(TIF)

S6 Fig Preliminary analysis reveals baseline signaling differences in signaling of responders vs non-responders to TNFi. Heatmap shows association of baseline signaling nodes with treatment response to TNFi. This was generated by unsupervised clustering analysis of treatment response of 33 autoantibody positive RA patients after 3 months of TNFi treatment in the univariate analysis controlling for age and baseline DAS28. The first seven columns represent unstimulated STAT3 signaling in: all lymphocytes; naive CD4+ T cells; CD4+ CD45RA+ T cells; all T cells; CD4+ CD45RA- T cells; CD4+ T cells; and central memory CD4+ T cells. The next two columns represent TNF stimulated signaling using Ikb in CD3- CD20- Lymphocytes (enriched for NK cells) using two different statistical matrics (Uu and log2fold metric). The next column shows IFNα stimulation with STAT3 readout in naive CD4− T cells. The final 7 columns represent IL-6 stimulated STAT3 in central memory CD4+ T cells and in naive CD4+ T cells; IL-6 stimulated STAT1 readout in central memory CD4- T cells (log2fold and Uu metric) and IL-6 stimulated STAT3 readout in B cells and memory B cells (log2fold and Uu metric). The fold metric measures the magnitude of the responsiveness, while Uu matric measures the fraction or proportion, of a cell population to modulation relative to the same cell population in the reference well. The Uu metric has an expected value of 0.5. A value different from 0.5 indicates the responsive population has shifted to higher fluorescence (values >0.5) or to lower fluorescence (values <0.5).
(TIF)

S1 Table Modulation conditions tested across samples/cohorts. (DOCX)

S2 Table Antibodies used in the studies. (DOCX)

S3 Table Phenotypic definition of cell populations analyzed in the studies. (DOCX)
