# Supplementary figures and images for: Diminished cytokine-induced Jak/STAT signaling is associated with rheumatoid arthritis and disease activity
Source: PLoS One. 2021 Jan 14;16(1):e0244187. doi: 10.1371/journal.pone.0244187 (PMC7808603; doi:10.1371/journal.pone.0244187)

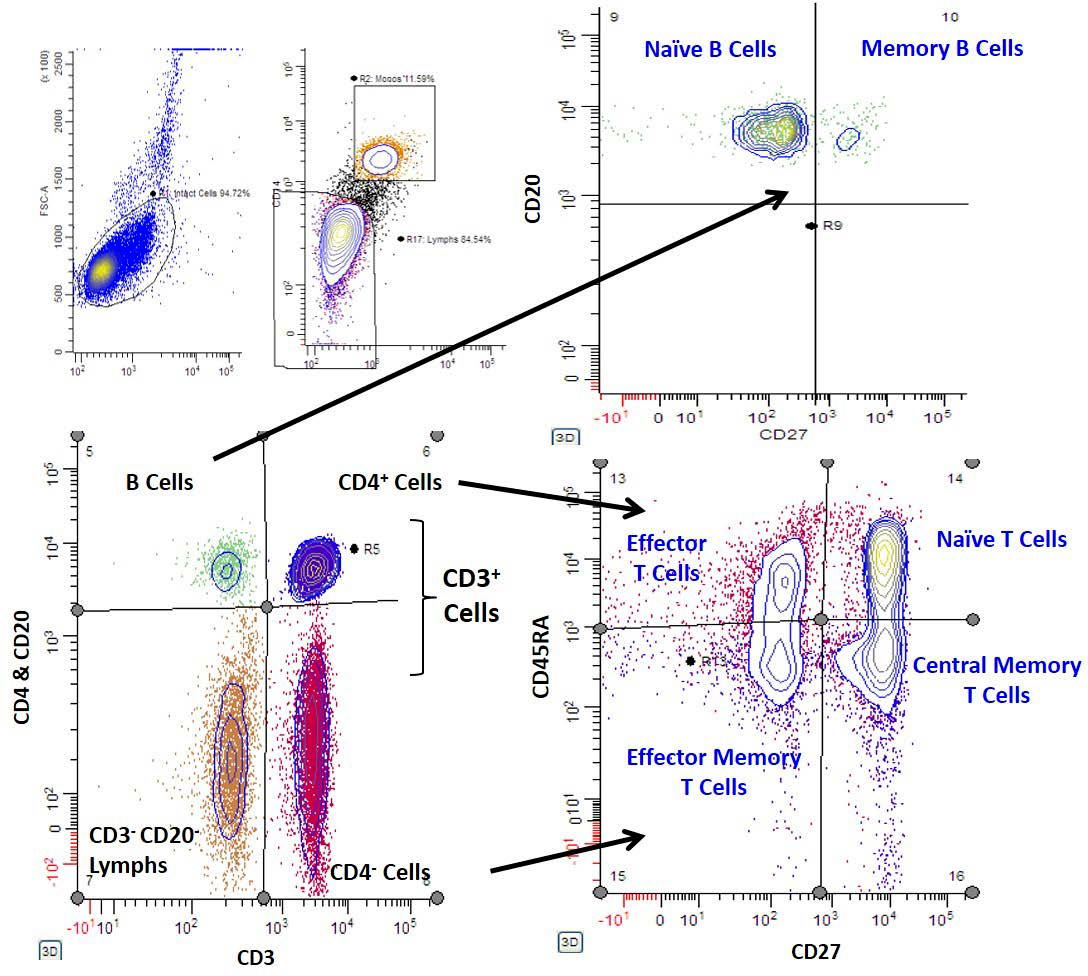

Supplement: S1 Fig — The gating strategy showed how to identify 21 cell subsets from PBMC with SCNP. Lymphocytes and monocytes were identified by forward scatter (FSC), side scatter (SSC). Non-viable cells were excluded based on the staining of propidium iodide (PI) staining (Cohort 1) or Amine Aqua viability dye (TT0, T6M). Lymphocytes were gated based on the expression of CD3, CD4, and CD20 to identify CD20+ B cells, CD3+CD4+ T helper cells, CD3+CD4- cytotoxic T cells, and CD3-CD20- lymphocytes that are predominantly NK cells. B cells were subdivided into naive (CD27-) and memory (CD27+) cell subsets based on CD27 expression. T helper and cytotoxic T cell populations were further subdivided into effector T cells (CD45RA+CD27-), naive T cells (CD45RA+CD27+), effector memory T cells (CD45RA-CD27-), and central memory T cells (CD45RA-CD27+). (TIF) [file pone.0244187.s001.tif]

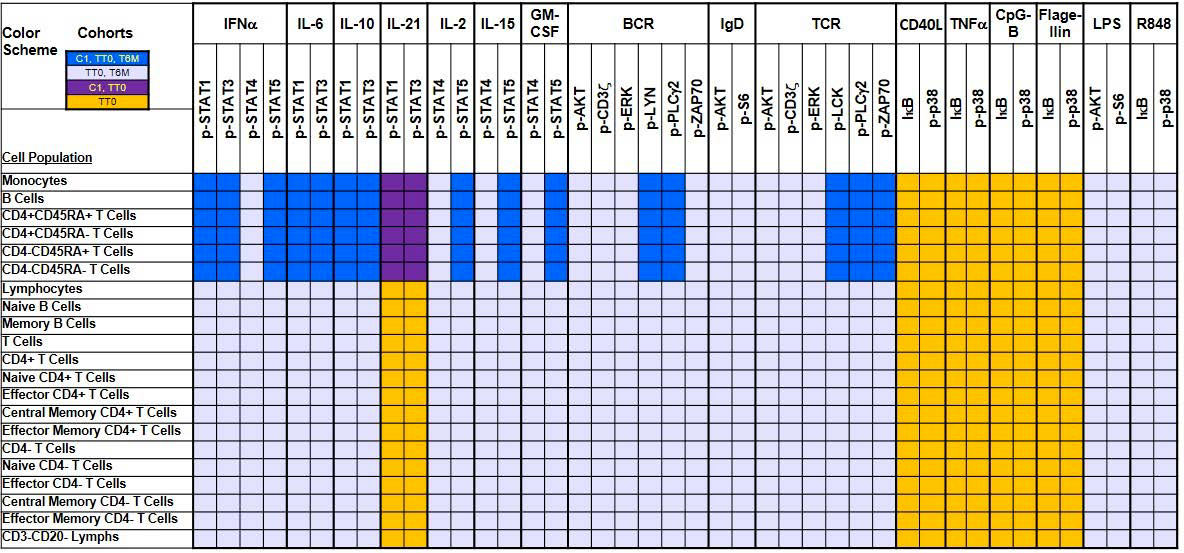

Supplement: S2 Fig — A total of 42 signaling nodes (modulator → readout) in 21 immune cell subsets were evaluated with the advantage of SCNP. A core set of nodes (15 in total) and cell populations (6 in total) were analyzed in all 3 sets of samples (dark blue). In addition, due to cells availability, analyses performed in TT0 only are highlighted in yellow, analyses performed in Cohort 1 and TT0 are labeled in purple, and analyses performed in TT0 and T6M are labeled in gray. The signaling pathways of peripheral blood cells from RA patients and HC were modulated using cytokines (IFNα, IL-2, IL-6, IL-10, IL-15, IL-21, GM-CSF), crosslinking antibodies to B and T cell receptors (BCR, TCR, IgD), and TLR agonists (CD40L, TNFα, Resiquimod R848), pathogen-associated molecules (CpG-B, Flagellin and LPS) as shown on the top row. The resulting readouts measured are shown on the second row, and cell subsets analyzed are shown in the left column. (TIF) [file pone.0244187.s002.tif]

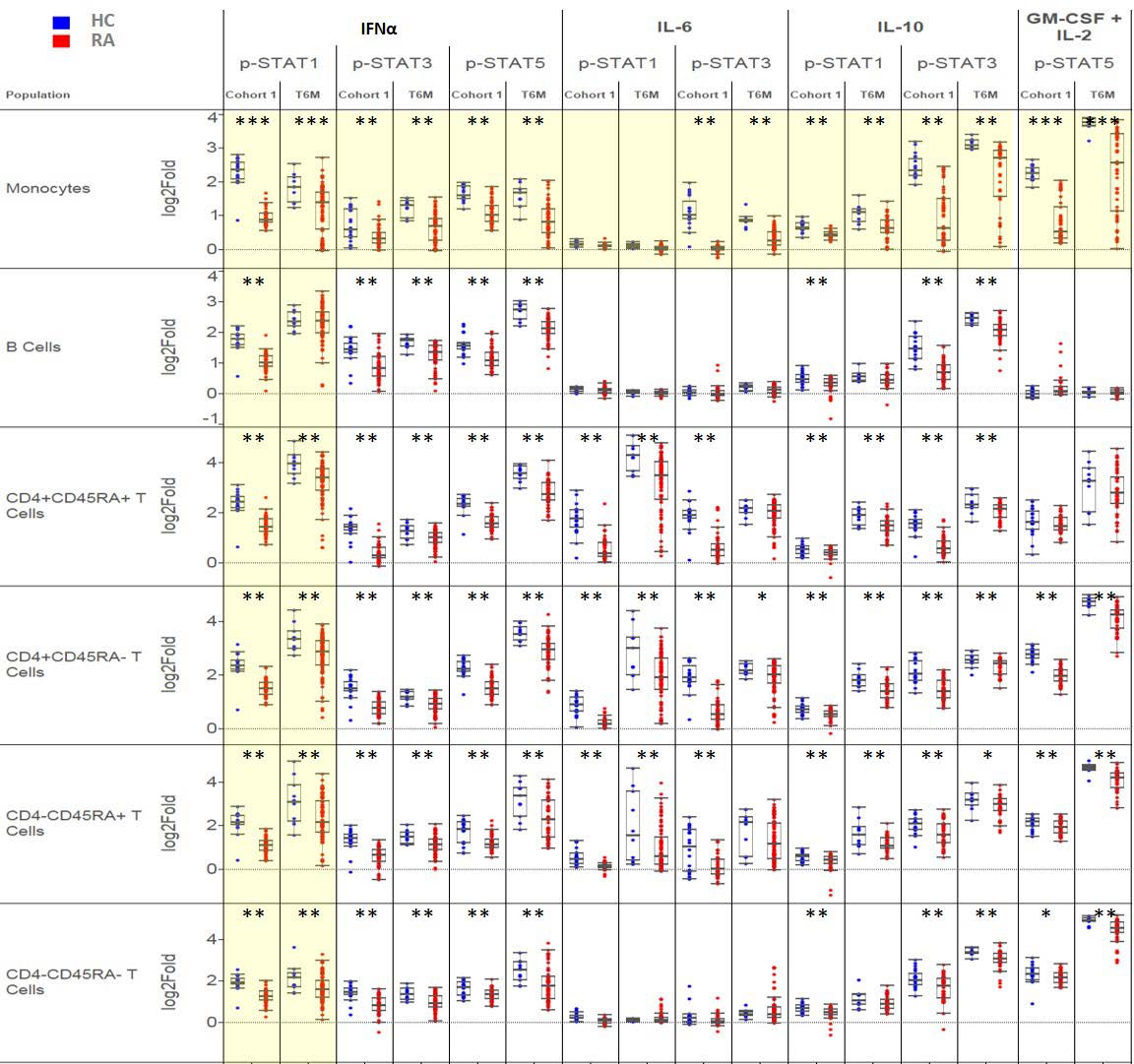

Supplement: S3 Fig — Analyses shaded in yellow are shown in detail in Fig 1C and 1D. * Differences between RA and HC were statistically significant (Wilcoxon signed-rank test) at p<0.05. ** Differences between RA and HC were statistically significant at p<0.01. *** Differences between RA and HC were statistically significant at p<0.001. (TIF) [file pone.0244187.s003.tif]

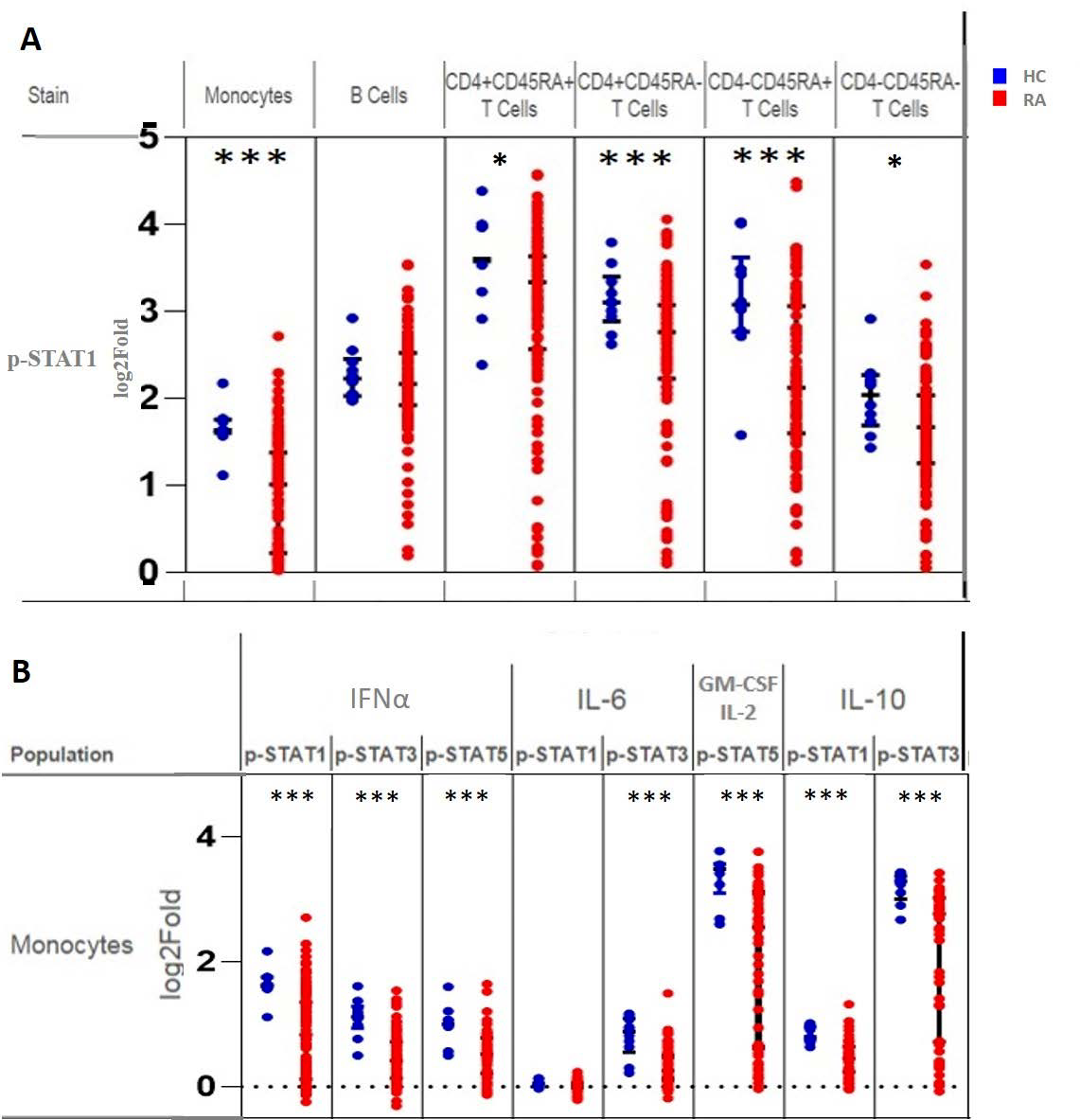

Supplement: S4 Fig — A. Significantly reduced IFNα→p-STAT1 signaling in 5 of 6 immune cell subsets of TT0 RA patients (n = 146) compared to HC (n = 10). * Differences between RA and HC were statistically significant (Wilcoxon signed-rank test) at p<0.05. *** Differences between RA and HC were statistically significant at p<0.001. B. Significantly reduced cytokine-induced signaling were found in monocytes of TT0 RA patients compared to HC, except IL6→p-STAT1. *** Differences between RA and HC were statistically significant at p<0.001. (TIF) [file pone.0244187.s004.tif]

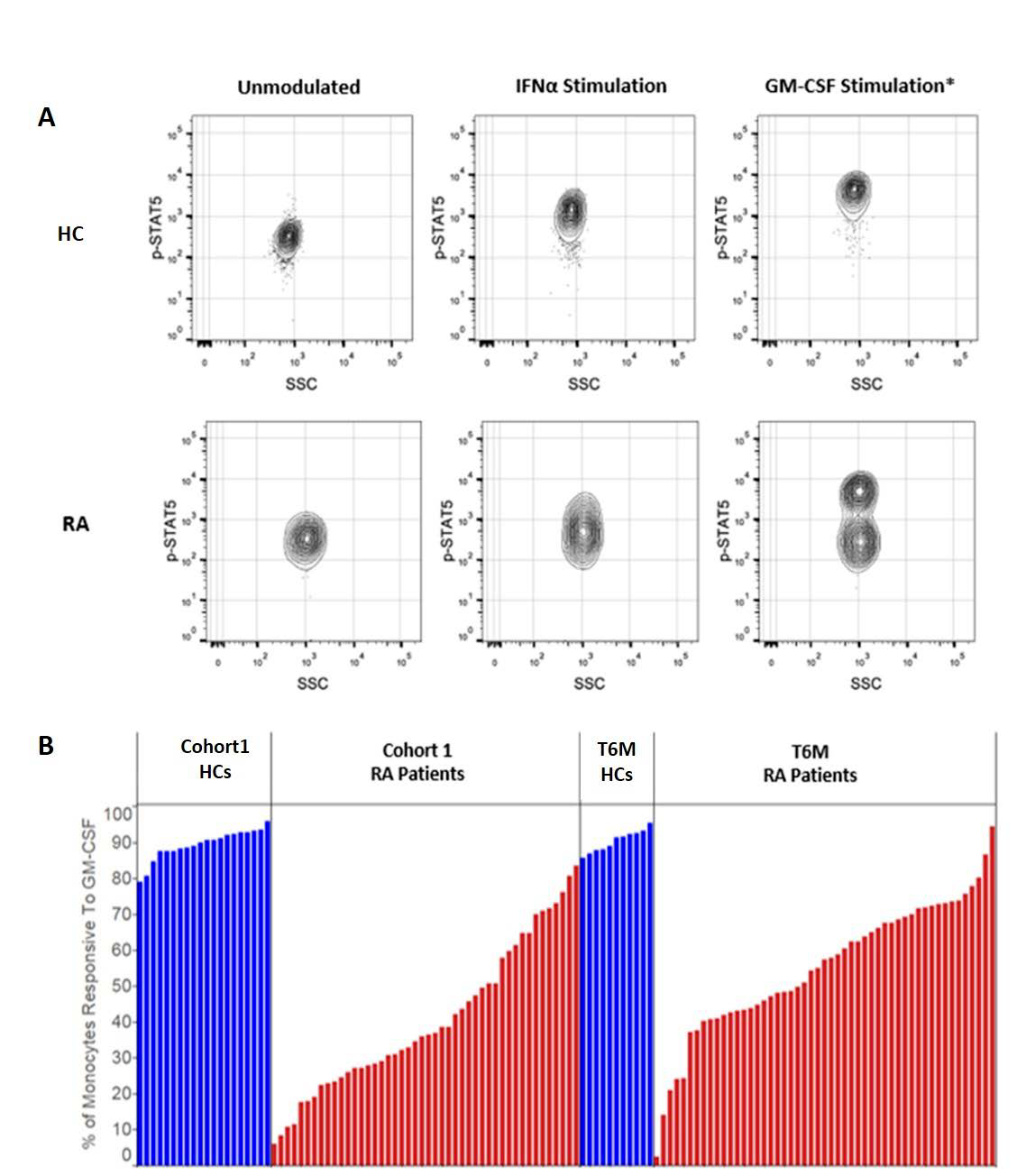

Supplement: S5 Fig — A. Representative contour plots show p-STAT5 in monocytes from one HC and from one RA patient under three different conditions: basal (unmodulated); IFNα stimulation, and GM-CSF + IL-2 stimulation. Monocytes from the RA patients showed a bimodal GM-CSF→p-STAT5 response whereas IFNα→p-STAT5 was unimodal. B. Histograms show percentages of monocytes that respond to GM-CSF from RA patients and HC. (TIF) [file pone.0244187.s005.tif]

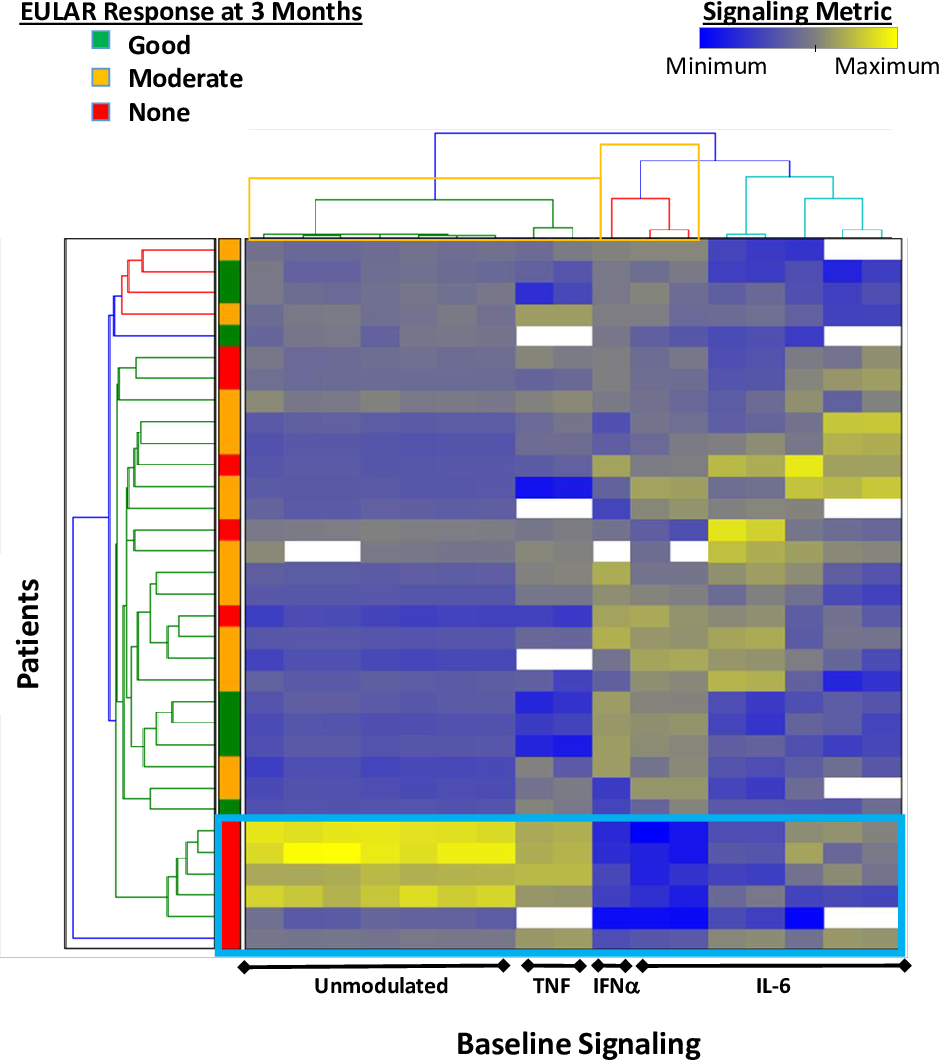

Supplement: S6 Fig — Heatmap shows association of baseline signaling nodes with treatment response to TNFi. This was generated by unsupervised clustering analysis of treatment response of 33 autoantibody positive RA patients after 3 months of TNFi treatment in the univariate analysis controlling for age and baseline DAS28. The first seven columns represent unstimulated STAT3 signaling in: all lymphocytes; naive CD4+ T cells; CD4+ CD45RA+ T cells; all T cells; CD4+ CD45RA- T cells; CD4+ T cells; and central memory CD4+ T cells. The next two columns represent TNF stimulated signaling using Ikb in CD3- CD20- Lymphocytes (enriched for NK cells) using two different statistical matrics (Uu and log2fold metric). The next column shows IFNα stimulation with STAT3 readout in naive CD4− T cells. The final 7 columns represent IL-6 stimulated STAT3 in central memory CD4+ T cells and in naive CD4+ T cells; IL-6 stimulated STAT1 readout in central memory CD4- T cells (log2fold and Uu metric) and IL-6 stimulated STAT3 readout in B cells and memory B cells (log2fold and Uu metric). The fold metric measures the magnitude of the responsiveness, while Uu matric measures the fraction or proportion, of a cell population to modulation relative to the same cell population in the reference well. The Uu metric has an expected value of 0.5. A value different from 0.5 indicates the responsive population has shifted to higher fluorescence (values >0.5) or to lower fluorescence (values <0.5). (TIF) [file pone.0244187.s006.tif]
