# Supplementary material for: Diminished cytokine-induced Jak/STAT signaling is associated with rheumatoid arthritis and disease activity
Source: PLoS One. 2021 Jan 14;16(1):e0244187. doi: 10.1371/journal.pone.0244187 (PMC7808603; doi:10.1371/journal.pone.0244187)
Supplement: S1 Table — (DOCX) [file pone.0244187.s007.docx]

**S1 Table.** Modulation conditions tested across samples/cohorts.

| **Modulator** | **Cohort 1** | **TT0 and T6M** |
| --- | --- | --- |
| IFNα | 10,000 IU/mL (Sigma) | 1,000 IU/mL (PBL Assay Science) |
| IL-6 | 50 ng/mL (BD) | 50 ng/mL (R&D Systems) |
| IL-10 | 50 ng/mL (Peprotech) | 50 ng/mL (BD) |
| IL-21 | 50 ng/mL (Peprotech) | 50 ng/mL (Peprotech) |
| IL-2 | 0.2 ng/mL and 50 ng/mL (BD) | 50 ng/mL (R&D Systems) |
| IL-15 | 50 ng/mL (Peprotech) | 50 ng/mL (Peprotech) |
| GM-CSF | 50 ng/mL (Peprotech) | 10 ng/mL (BD) |
| BCR modulation | 10 μg/mL each of anti-IgG (BD), anti-IgM (BD) | 20 μg/mL anti-IgM (Southern Biotech) |
| Anti-IgD | - | 5 μg/mL (BD) |
| TCR modulation | 10 μg/mL each of anti-CD3e (BD) and anti-mouse (Santa Cruz Biotechnology) | 3 μg/mL anti-CD3e (eBioscience), 10 μg/mL anti-mouse (Santa Cruz Biotechnology) |
| CD40L | - | 0.5 μg/mL (R&D) |
| TNFα | - | 100 ng/mL (BD) |
| CpG-B | - | 5 μg/mL |
| Flagellin | - | 10 μg/mL (Invivogen) |
| LPS | - | 1 μg/mL (Sigma) |
| R848 | - | 5 μg/mL(Invivogen) |
