# Supplementary material for: Diminished cytokine-induced Jak/STAT signaling is associated with rheumatoid arthritis and disease activity
Source: PLoS One. 2021 Jan 14;16(1):e0244187. doi: 10.1371/journal.pone.0244187 (PMC7808603; doi:10.1371/journal.pone.0244187)
Supplement: S2 Table — (DOCX) [file pone.0244187.s008.docx]

**S2 Table.** Antibodies used in the studies.

| **Antibody** | **Cohort 1** | **TT0 and T6M** |
| --- | --- | --- |
| CD14 | - | RMO52 (Beckman Coulter) |
| CD19 | HIB19 (eBioscience) | HIB19 (BD) |
| CD20 | - | H1 (BD) |
| CD27 | - | L128 (BD) |
| CD3 | UCHT1 (Invitrogen), UCTH1 (BD) | UCHT1 (BD) |
| CD33 | P67.6 (BD) | - |
| CD4 | RPA-T4 (BD) | RPA-T4 (BD) |
| CD45RA | HI100 (eBioscience) | HI100 (BD) |
| Cleaved PARP (D214) | - | F21-852 (BD) |
| IgD | - | IA6-2 (BD) |
| IgM | - | G20-127 (BD) |
| IκB | - | L35A5 (Cell Signaling Technologies) |
| p-AKT(S473) | - | 193H12 (Cell Signaling Technologies) |
| p-CD3ζ(Y142) | - | K25-407.69 (BD) |
| p-ERK(T202/Y204) | - | D13.14.4E (Cell Signaling Technologies) |
| p-LCK(Y505) | 4/LCK-Y505 (BD) | 4/LCK-Y505 (BD) |
| p-p38(T180/Y182) | - | 36/p38 (pT180/pY182 (BD) |
| p-PLCγII | K86-689.37 (BD) | K86-689.37 (BD) |
| p-S6 | - | 2F9 (Cell Signaling Technologies) |
| p-STAT1(Y701) | 4a (BD) | 4a (BD) |
| p-STAT3(Y705) | 4/P-STAT3 (BD) | 4/P STAT3 (BD) |
| p-STAT4(Y693) | - | 38/p-STAT4 (BD) |
| p-STAT5 | 47/STAT5(pY694) (BD) | 47/STAT5(pY694) (BD) |
| p-Zap70(Y319)/p-Syk(Y352) | 17A/P-ZAP70 (BD) | 17A/P-ZAP70 (BD) |
