# Supplementary material for: Diminished cytokine-induced Jak/STAT signaling is associated with rheumatoid arthritis and disease activity
Source: PLoS One. 2021 Jan 14;16(1):e0244187. doi: 10.1371/journal.pone.0244187 (PMC7808603; doi:10.1371/journal.pone.0244187)
Supplement: S3 Table — (DOCX) [file pone.0244187.s009.docx]

**S3 Table.** Phenotypic definition of cell populations analyzed in the studies.

| **Cell Population** | **Definition** | **Cohort** | **TT0, T6M** |
| --- | --- | --- | --- |
| Monocytes | CD14+ or CD33+, high SSC | X | X |
| Lymphocytes | CD14- or CD33-, low SSC | X | X |
| B cells | CD19+ or CD20+ lymphocytes | X | X |
| Naïve B cells | CD20+CD27- lymphocytes | - | X |
| Memory B cells | CD20+CD27+ lymphocytes | - | X |
| T cells | CD3+ lymphocytes | X | X |
| CD4+ T cells | CD3+CD4+ lymphocytes | X | X |
| CD4+CD45RA+ T cells | CD3+CD4+CD45RA+ lymphocytes | X | X |
| Effector CD4+ T cells | CD3+CD4+CD45RA+CD27- lymphocytes | - | X |
| Naïve CD4+ T cells | CD3+CD4+CD45RA+CD27+ lymphocytes | - | X |
| Effector Memory CD4+ T cells | CD3+CD4+CD45RA-CD27- lymphocytes | - | X |
| Central Memory CD4+ T cells | CD3+CD4+CD45RA-CD27+ lymphocytes | - | X |
| CD4- T cells | CD3+CD4- lymphocytes | X | X |
| CD4-CD45RA+ T cells | CD3+CD4-CD45RA+ lymphocytes | X | X |
| Effector CD4- T cells | CD3+CD4-CD45RA+CD27- lymphocytes | - | X |
| Naïve CD4- T cells | CD3+CD4-CD45RA+CD27+ lymphocytes | - | X |
| CD4-CD45RA- T cells | CD3+CD4-CD45RA- lymphocytes | X | X |
| Effector Memory CD4- T cells | CD3+CD4-CD45RA-CD27- lymphocytes | - | X |
| Central Memory CD4- T cells | CD3+CD4-CD45RA-CD27+ lymphocytes | - | X |
| CD3-CD20- Lymphocytes | CD3-CD20- lymphocytes | - | X |
